# A Comparative Study of Elective Sublay Versus Onlay Repair for Non-complex, Small, and Medium-Size Incisional Hernia: Post-operative Complications in a Tertiary Hospital in Ranchi, India

**DOI:** 10.7759/cureus.59593

**Published:** 2024-05-03

**Authors:** Farrukh Hassan, Kumar Gaurav, Krishan Kumar, Kamlesh Kumar, Balamurali B., Venkatesh N., Muklesh K Mehta, Praveenkumar A.

**Affiliations:** 1 General Surgery, Rajendra Institute of Medical Sciences, Ranchi, IND; 2 Community Medicine, Rajendra Institute of Medical Sciences, Ranchi, IND

**Keywords:** ranchi, recurrence, postoperative outcome, sublay, onlay, incisional hernia

## Abstract

Background: The most difficult hernia surgery is the repair of the ventral hernia, which is caused by aberrant organ or tissue protrusions through the abdominal wall. Factors like obesity, smoking, and chronic medical conditions contribute to their formation. Surgical strategies have evolved from anatomical repair to mesh hernioplasty, with mesh placement playing a significant role in outcomes. The ideal anatomical location for mesh placement remains debated due to varying results. So, the objective of the study is to compare early postoperative complications, surgical site infection, and incidence of recurrence between sublay and onlay mesh placement repair of incisional hernias of <10 cm in diameter, at a tertiary hospital in Ranchi.

Methods: This retrospective comparative study was conducted over a period of January 2022 to January 2024 at the Rajendra Institute of Medical Science, Ranchi, India. During the study period, 96 patients were operated on, and their demographic details, along with their position of mesh placement and postoperative complications (seroma formation, wound infection, postoperative hospital stays, and recurrence), were retrieved from the hospital data. Comparisons between onlay and sublay groups in terms of post-operative complications were made.

Results: Within the study period, a total of 96 patients were operated on for incisional hernia. In this study, 36 (37.5%) were male and 60 (62.5%) were female, with a male-to-female ratio of 0.6:1. Out of the total number of patients, 56 (58.4%) had a past history of emergency surgery. It was observed that there was a higher incidence of seroma formation in the onlay group compared to the sublay with a statistical significance p-value of 0.027. The incidence of wound infection was found to be statistically significant (p-value = 0.035) between the onlay and sublay groups. In a period of six-month follow-up, three patients of the total study population had an incidence of recurrent incisional hernia, of which two from the onlay group and one from the sublay group were present, and there was no statistical significance (p-value > 0.5).

Conclusions: Based on our retrospective analysis, we can say that there is a lower incidence of postoperative complications and recurrence in sublay repair, along with a shorter postoperative hospital stay, making it a preferred method of repair over onlay.

## Introduction

Of all the hernia surgeries done, the most difficult and challenging is the ventral hernia repair, which is an abnormal protrusion of an organ or tissue through the anterior abdominal wall [1,2]. They are divided into spontaneous and acquired; for the latter, expertise is required for the repair [2]. As there is an increasing trend in exploratory laparotomy, there is a high incidence of incisional hernia [3], which is around 2-11% to 10-20% [4,5]. This hernia can occur anywhere at the site of a previous surgical scar through the fascial defect, and its formation may start around the early postoperative days. The healed skin masks the initial stage [1].

Apart from the technique used for previous operations and wound infections, associated factors like obesity, smoking, and chronic medical conditions like diabetes and COPD all lead to the formation of incisional hernias [6-8].

The operative strategies used for the correction of incisional hernia have gradually progressed from anatomical repair, which has shown a high recurrence rate (31-49%), to mesh hernioplasty with less recurrence rate (≤10%) [6]. There are many studies that suggest that the position of the mesh has a major part in the outcome [9], because of postoperative complications like wound infection, seroma formation, and recurrence rate. The complication not only affects the physical and mental health of both the patient and surgeon but also carries a huge resource loss in managing the complication [5,10].

Onlay repair is the placement of mesh above the anterior rectus sheath and beneath the subcutaneous tissue. Sublay repair is the process wherein the mesh is positioned either beneath the retro-muscular or preperitoneal space [2,11].

Since some of the studies have shown that the sublay technique has the edge over the onlay technique [9] and other studies have found no significant reduction in the incidence of complications [7], the ideal anatomical location for mesh placement is still in debate. So in this study, we have done a retrospective study of incisional hernia repair at Rajendra Institute of Medical Sciences (RIMS), Ranchi, India, between 2021 and 2023. To compare early postoperative complications, surgical site infection, and incidence of recurrence between sublay and onlay mesh placement repair of incisional hernias of <10 cm in diameter.

## Materials and methods

This retrospective comparative study was conducted over a period of January 2022 to January 2024 at the RIMS, Ranchi, India. The inclusion criteria for the study are patients aged over 18 with American Society of Anesthesiologists (ASA) grade I & II, a hernia defect less than 10 cm, undergoing elective incisional hernia repair (onlay or sublay), and followed postoperatively for six months. The exclusion criteria are morbid obesity (body mass index (BMI) > 40 kg/m^2^), recurrent incisional hernia repair, chronic obstructive pulmonary disease, cardiovascular disease, being immunocompromised, having a history of immunosuppressive drug intake, or a history of any malignancy.

S = z2*p(1-p)/e2 (S is the sample size, z is the statistic for a degree of confidence, p is proportion, and e is the margin of error) was the formula used to determine the sample size. Given z = 1.96 (with a 95% confidence interval (CI), p = 0.2, and e = 0.05, S = 222 may be found. The formula S' = S/(1+S/N) was used to calculate the new sample size (S') given a limited population size (N). Here, N = 90 represents the total number of patients treated for incisional hernias at RIMS in 2021-2022. Thus, S' = 64, but 96 patients received surgery and satisfied the inclusion criteria during the study period.

After getting approval from the Institutional Ethics Committee of RIMS, Ranchi (approval no. 191), all the information like age, sex, BMI, previous operation (elective or emergency), current repair technique (onlay or sublay), postoperative outcomes (seroma formation, hematoma formation, wound infection, total number of days drain placed, visual analog score (VAS) for postoperative pain), postoperative hospital stay, and any readmission for recurrence were retrieved from hospital records.

Data analysis

The data collected was entered into a Microsoft Excel spreadsheet (student version 2021, Microsoft Corporation, Redmond, United States) and then exported to the data editor of IBM SPSS Statistics for Windows, Version 27 (Released 2020; IBM Corp., Armonk, New York, United States) for analysis. Continuous variables were expressed as mean ± SD, and the unpaired t-test was the statistical tool used. They are age, BMI, number of days drain placed, postoperative pain score, and postoperative stay. Categorical variables were summarized as frequencies and percentages. They were age group, gender, previous operation (elective or emergency), seroma formation, wound infection, and recurrence. To evaluate the categorical variables, the chi-square test was used as a statistical tool for the study.

## Results

**Figure 1:**
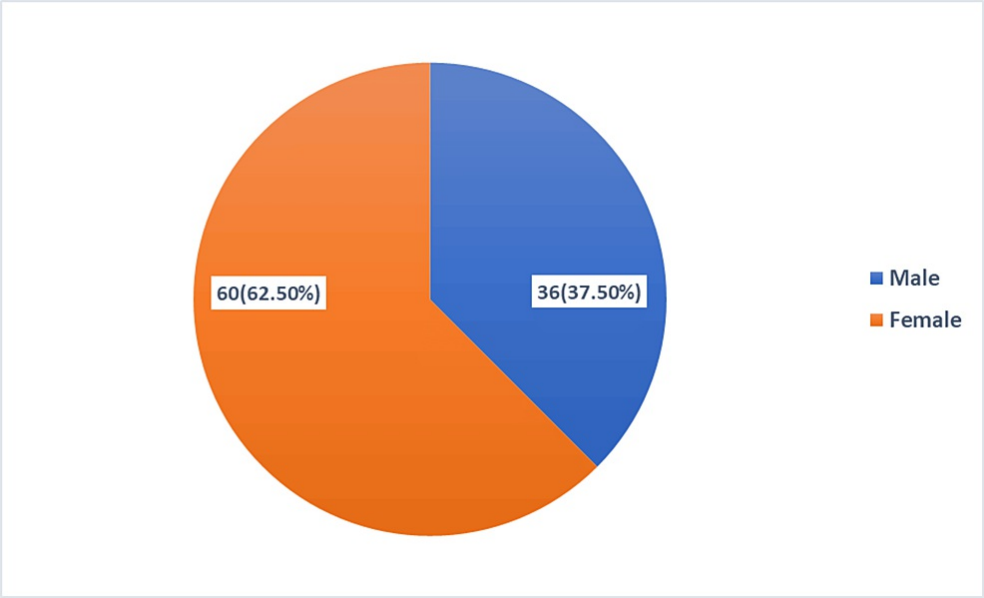
Incidence of incisional hernia according to gender

A total of 96 individuals had incisional hernia operations during the research period. The study's male-to-female ratio was 0.6:1, with 36 (37.5%) men and 60 (62.5%) women (Figure 1).

**Figure 2:**
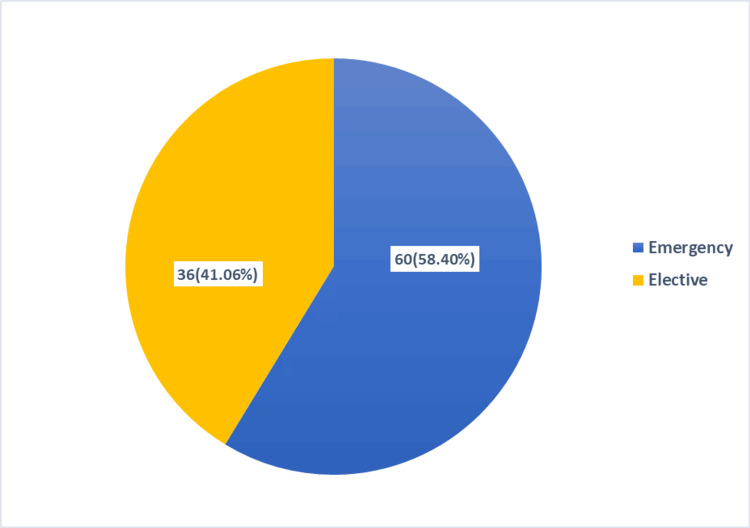
Incidence of incisional hernia according to previous operation

The mean age for incisional hernia in our study population is 45.4 ± 12.29. Out of the total 96 patients, 56 (58.4%) had a past history of emergency surgery, and 40 (41.6%) had elective surgery (Figure 2).

In this retrospective study, onlay mesh repair was done in 50 patients and sublay in 46 patients. The mean BMI in the onlay group was 27.7 ± 3.88, while in the sublay group, it was 25.7 ± 3.14. The mean intra-operative time taken for onlay and sublay repair was 88.1 ± 31.39 and 104.9 ± 39.41 minutes, respectively. It was found to be statistically significant by an unpaired t-test (Table 1).

**Table 1:** An analysis of intra-operative time for each group

Groups	Intra-operative time in minutes (mean ± SD)
Onlay	88.1 ± 31.39
Sublay	104.9 ± 39.41
p-value	0.0225

The mean post-operative pain determined by the VAS between the onlay and sublay was 5.18 ± 1.1 and 5.02 ± 1.08, respectively. Severe pain (VAS > 5) was observed more in onlay group 19 (38%), in contrast to sublay group 13 (28.1%), but it was not statistically significant by unpaired t-test (Table 2).

**Table 2:** Analysis of pain following surgery between groups VAS: visual analog score

Groups	VAS > 5	VAS ≤ 5	p-value
Sublay	13 (28.26%)	33 (71.73%)	0.311
Onlay	19 (38%)	31 (62%)	

Post-operative outcomes like seroma formation and wound infection were assessed among onlay and sublay groups. With a statistical significance of p-value = 0.027, it was found that the onlay group had a higher incidence of seroma development than the sublay. The incidence of wound infection was statistically significant (p-value = 0.035) between the onlay and sublay groups (Table 3).

**Table 3:** Post-operative complication between groups

Variables		Onlay		Sublay		p-value
		N	%	N	%	
Seroma	Present	16	72.70%	6	27.30%	0.027
	Absent	34	45.90%	40	54.10%	
Wound infection	Present	9	81.18%	2	18.18%	0.035
	Absent	41	48.23%	44	51.76%	

In comparison to the onlay group, the sublay group's mean drain placement length in days (mean ± SD) was shown to be substantially shorter (Table 4).

**Table 4:** Analysis of duration of drain placement between groups

Groups	Duration of drain placement in days (mean ± SD)
Onlay	5.08 ± 1.88
Sublay	4.08 ± 1.83
p-value	0.0102

The sublay group's mean hospital stay following surgery in days (mean ± SD) was found to be considerably lower than the onlay groups (Table 5).

**Table 5:** Post-operative hospital stays between groups

Groups	Post-operative hospital stays in days (mean ± SD)
Onlay	9.38 ± 4.46
Sublay	7.8 ± 2.66
p-value	0.0125

In a period of six-month follow-up, three patients of the total study population had an incidence of recurrent incisional hernia, of which two from the onlay group and one from the sublay group were present, and no statistical significance (p-value > 0.5) was observed (Table 6).

**Table 6:** Comparing the incidence of recurrence between the two groups

Group	Recurrence		Total	p-value
	Present	Absent		
Onlay	2 (4%)	48 (96%)	50	0.607
Sublay	1 (2.17%)	45 (97.82%)	46	

## Discussion

Incisional hernia, as the name suggests, occurs through the previous surgical scar and can occur anywhere in the anterior abdominal wall [12]; its incidence is in the range of 2-11% to 10-20% [4]. The risk of developing incisional hernia is high in patients with a history of previous surgical site infection [7], tension closure of the abdominal wall, and other factors like obesity, smoking, and diabetes [6].

Aseptic surgical field, healthy wound edge, using a suture-to-wound length ratio of 4:1 to maintain minimal tension in the suture line, using monofilament slowly absorbable suture material, and surgical technique (small stitches, small bites) are recommended for the prevention of incisional hernia [1,13].

One of the most difficult surgical procedures carried out in clinical practice is the correction of an incisional hernia. Technical expertise from a surgeon is required for a better outcome. There are many surgical strategies available for incisional hernia repair, like open anatomical closure, open mesh hernioplasty, and laparoscopic mesh hernioplasty. Due to the low incidence of recurrence shown by mesh hernioplasty, it is considered a standard operation for incisional hernia as it provides a tension-free closure of the defect and increases the tensile strength of the wound [1,9,14]. Mesh repair works on the principle of Laplace’s law by distributing the pressure across the mesh [8]. The anatomical position of the mesh is an important aspect in the development of complications in the early post-operative recovery period and the development of recurrence [4].

In our retrospective study, 96 patients had undergone incisional hernia repair during the study period. The incidence of incisional hernia had a female predominance of 60 (62.5%), with a male-to-female ratio of 0.6:1. This finding is compatible with the studies of Kumar et al., Saeed et al., Raghuveer et al., and Kumar et al. [3-6]. This may be because females are more exposed to lower midline incisions, and overstretching of muscles during pregnancy, along with aging, causes laxity of muscle [3]. The mean age for the development of incisional hernia according to our study is 45.4 ± 12.29, which is similar to the study conducted by Naz et al., Saeed et al., Khawaja et al., Gondal and Anjum, and Kumar et al. [4,6,10,12,15]. Out of the total 96 patients, 56 (58.40%) with incisional hernia had a previous history of emergency surgical procedures; this might be the consequence of surgical site infection followed by poor wound healing [6].

The mean intra-operative time taken for onlay and sublay techniques is 88.1 ± 31.39 and 104.9 ± 39.41 minutes, respectively, and it is statistically significant (p < 0.05). Our observation is compatible with Raghuveer et al., Shekhar et al., and Venclauskas et al. [5,16,17]. The reason for the longer intra-operative time observed in the sublay technique is due to dissecting the retro-muscular plane while securing the hemostasis at the same time. It also depends on the experience of the surgeon operating [5].

Post-operative pain determined by the VAS after 48 hours of operation between the onlay and sublay groups was not statistically significant (p-value > 0.05). Some studies have reported that immediate post-operative pain was significantly lower among patients in the sublay group Shekhar et al. and Saeed et al. [4,16]. A study done by Kumar et al. has shown that patients who underwent sublay repair experienced more pain in the postoperative period [3].

In our retrospective study, the occurrence of seroma formation post-operatively is found to be significantly high (p-value < 0.05) in the onlay group. The onlay procedure frequently results in seroma development, according to multiple research Demetrashvili et al., Saeed et al., Raghuveer et al., Venclauskas et al., and Ibrahim et al. [4,5,14,17,18], but Kumar et al. [3] showed no significance. When onlay repair mesh is placed below the subcutaneous plane, this may initiate foreign body reactions and fluid collection, which leads to seroma formation [18].

When the incidence of wound infection was compared between the groups, two patients in the sublay group and nine patients in the onlay group had wound infections, there was statistical significance (p-value < 0.05) between the groups. Out of the total population, 11 patients had wound infections, of which 9 (81.18%) were in the onlay group. According to other scientific literature by Gondal and Anjum, Naz et al., Saeed et al., Venclauskas et al., Khawaja et al., Raghuveer et al., and Kumar et al. [3-5,10,12,15,17], there is a high wound infection rate in the onlay group.

In this study, there is a statistically significant (<0.05) lower mean duration of drain placement in the sublay group; comparable results were observed in studies by Raghuveer et al. and Naz et al. [5,15]. A lower mean postoperative hospital stay in days was noted in the sublay group (7.8 ± 2.66) compared with the onlay group (9.38 ± 4.46), which is statistically significant (p-value < 0.05). This finding is compatible with the studies by Shekhar et al., Saeed et al., Gondal and Anjum, Ahmed and Mehboob, and Raghuveer et al. [4,5,12,16,19].

One of the complications that is stressful for both patient and surgeon is the incisional hernia recurrence. It is an economic and psychological burden not only for the patient but also for the surgeon. Although, after the implementation of mesh repair, the rate of recurrence has come down drastically. Three patients in our study experienced recurrences; two of them belonged to the onlay group and one to the sublay group. Regarding the incidence of recurrence, the two groups did not differ statistically significantly. Shekhar et al., Demetrashvili et al., and Raghuveer et al. found similar results in the incidence of recurrence [5,14,16]. The Timmermans et al. study showed that mesh placed in the retro-muscular space had better outcomes due to high collagen deposition and high tensile strength [9].

Outcomes of emergency and laparoscopic incisional hernia repair, management of recurrent incisional hernia, short duration of follow-up, and smaller sample size of our study population were some of our limitations. In this study, potential biases include selection bias, as patients were not randomly assigned to sublay and onlay, leading to differences in baseline characteristics between the groups. Additionally, there is information bias, as some patients could not be included in the study due to a lack of crucial data in their medical records. Finally, there could be bias related to the surgeon's preference and experience for one technique over the other, influencing outcomes.

## Conclusions

Based on our retrospective analysis, we can say that there is a lower incidence of postoperative complications such as seroma development, wound infection, and recurrence in sublay repair. Sublay repair also has the advantage of a shorter postoperative hospital stay, making it a preferred method of repair over onlay. We recommend that a randomized controlled trial provide more scientific evidence to determine the efficiency of sublay and onlay techniques.
